# Novel Natural Products for Healthy Ageing from the Mediterranean Diet and Food Plants of Other Global Sources—The MediHealth Project

**DOI:** 10.3390/molecules23051097

**Published:** 2018-05-06

**Authors:** Birgit Waltenberger, Maria Halabalaki, Stefan Schwaiger, Nicolas Adamopoulos, Noureddine Allouche, Bernd L. Fiebich, Nina Hermans, Pidder Jansen-Dürr, Victor Kesternich, Luc Pieters, Stefan Schönbichler, Alexios-Leandros Skaltsounis, Hung Tran, Ioannis P. Trougakos, Alvaro Viljoen, Jean-Luc Wolfender, Christian Wolfrum, Nikos Xynos, Hermann Stuppner

**Affiliations:** 1 Institute of Pharmacy/Pharmacognosy and Center for Molecular Biosciences Innsbruck (CMBI), University of Innsbruck, 6020 Innsbruck, Austria; stefan.schwaiger@uibk.ac.at (S.S.); hermann.stuppner@uibk.ac.at (H.S.); 2 Department of Pharmacognosy and Natural Products Chemistry, Faculty of Pharmacy, National and Kapodistrian University of Athens, 15771 Athens, Greece; mariahal@pharm.uoa.gr (M.H.); skaltsounis@pharm.uoa.gr (A.-L.S.); 3 Department of New Business Development, Galenica SA, 14564 Kiffisia, Greece; nbd@galenica.gr; 4 Laboratory of Organic Chemistry, Natural Substances Team, Faculty of Sciences of Sfax, University of Sfax, PB 1171, 3000 Sfax, Tunisia; noureddineallouche@yahoo.fr; 5 Department of Research and Development, VivaCell Biotechnology GmbH, 79211 Denzlingen, Germany; fiebich@vivacell.de; 6 Natural Products and Food Research and Analysis (NatuRA), Department of Pharmaceutical Sciences, University of Antwerp, 2610 Antwerp, Belgium; nina.hermans@uantwerpen.be (N.H.); luc.pieters@uantwerpen.be (L.P.); 7 Institute of Biomedical Aging Research and CMBI, University of Innsbruck, 6020 Innsbruck, Austria; pidder.jansen-duerr@uibk.ac.at; 8 Department of Chemistry, Catholic University of the North, Casilla, Antofagasta 1280, Chile; vkestern@ucn.cl; 9 Department of Analytical Research, Bionorica research GmbH, 6020 Innsbruck, Austria; stefan.schoenbichler@bionorica.at; 10 Department of Pharmacognosy, Faculty of Pharmacy, University of Medicine and Pharmacy, Ho Chi Minh City 700000, Vietnam; tranhung@uphcm.edu.vn; 11 Department of Cell Biology and Biophysics, Faculty of Biology, National and Kapodistrian University of Athens, 15784 Athens, Greece; itrougakos@biol.uoa.gr; 12 Center for Natural Products in Drug Development, Department of Pharmaceutical Sciences, Tshwane University of Technology, Pretoria 0001, South Africa; viljoenam@tut.ac.za; 13 Phytochemistry and Bioactive Natural Products and Pharmacognosy, School of Pharmaceutical Sciences, EPGL, University of Geneva, University of Lausanne, 1211 Geneva 4, Switzerland; jean-luc.wolfender@unige.ch; 14 Department of Health Science and Technology, Laboratory of Translational Nutrition Biology, Swiss Federal Institute of Technology (ETH) Zürich, 8603 Schwerzenbach, Switzerland; christian-wolfrum@ethz.ch; 15 Department of Research and Development, Rousselet Centrifugation SA, 07100 Annonay, France; nxynos@kromaton.com

**Keywords:** MediHealth, healthy ageing, Mediterranean diet, food plants, natural products, metabolites, bioavailability, pharmacology, phytochemistry, synthetic chemistry

## Abstract

There is a rapid increase in the percentage of elderly people in Europe. Consequently, the prevalence of age-related diseases will also significantly increase. Therefore, the main goal of MediHealth, an international research project, is to introduce a novel approach for the discovery of active agents of food plants from the Mediterranean diet and other global sources that promote healthy ageing. To achieve this goal, a series of plants from the Mediterranean diet and food plants from other origins are carefully selected and subjected to in silico, cell-based, in vivo (fly and mouse models), and metabolism analyses. Advanced analytical techniques complement the bio-evaluation process for the efficient isolation and identification of the bioactive plant constituents. Furthermore, pharmacological profiling of bioactive natural products, as well as the identification and synthesis of their metabolites, is carried out. Finally, optimization studies are performed in order to proceed to the development of innovative nutraceuticals, dietary supplements or herbal medicinal products. The project is based on an exchange of researchers between nine universities and four companies from European and non-European countries, exploiting the existing complementary multidisciplinary expertise. Herein, the unique and novel approach of this interdisciplinary project is presented.

## 1. Introduction

Current demographic trends suggest a rapid increase in the percentage of elderly people in Europe for the coming decades, due to an increase in life expectancy and consistently low fertility levels [1]. Moreover, according to the World Health Organization (WHO), not only in Europe, but in populations around the world, there is a dramatic increase in the proportion as well as the absolute number of older people [2]. It is estimated that by 2050, the global population aged 60 years and over will double and reach 2 billion (22% of the world’s total population) [3]. Increasing life expectancy of the general population bears the inherent risk of an increase in physical and mental impairments, as well as in age-related diseases (e.g., cancer, neurodegeneration, metabolic disorders, etc.), posing a large and ever growing burden on our societies if we fail to develop appropriate counteracting strategies. In this situation, the concept of healthy ageing, i.e., a strategy that allows people to grow old in good health, becomes very important and was recognized as one of the most critical goals by the WHO [2].

Many theories have attempted over the years to explain in pertinent ways the complex set of events leading to ageing and degeneration. The free radical theory of ageing suggests that ageing could be attributed to deleterious effects of gradually accumulating reactive oxygen species (ROS) on various cellular components. The ubiquitous nature of ROS targets (e.g., lipids, proteins, or DNA) explains the large array of stochastic damage that these molecules bring about in any living organism, causing a progressive functional deterioration of cells, tissues, and organ systems [4]. Two major intracellular sources of ROS in human cells are mitochondria and NADPH oxidases, accounting for the production of distinct ROS in certain subcellular compartments. Whereas our understanding of the underlying biology is far from complete, it seems that different ROS species originating from different sources can have quite different effects on cell physiology. Damages elicited on cellular biomolecules by unbalanced ROS and most likely other pathways lead to the progression of the so called age-related diseases that affect both lifespan as well as life quality [5]. In order to ensure homeostasis and prevent unbalanced ROS-mediated cellular destabilization, cells have evolved a complex and interconnected network of sensors and downstream antioxidant responses. The NF-E2-related factor-2 (Nrf2)/Kelch-like ECH-associated protein 1 (Keap1) signaling pathway is central to cellular responses to oxidative and electrophilic stress [6]. In non-stressful conditions, the Nrf2 is retained in the cytoplasm by the actin-binding Keap1 and it is targeted for degradation by the proteasome. Under conditions of oxidative stress (i.e., accumulation of ROS beyond a physiological level), the Nrf2-Keap1 interaction is disrupted, allowing Nrf2 to translocate to the nucleus where, by binding to antioxidant response elements (AREs), it stimulates the expression of phase II and antioxidant enzymes. As was recently shown [7,8], loss of proteasome function signals to an age- and tissue-dependent feedback regulatory circuit aiming to restore proteostasis and prevent premature ageing. Therefore, it has been proposed that either inhibition of extensive ROS accumulation (e.g., by inhibiting NADPH oxidase 4 (Nox4) enzymes) or activation of Nrf2 and/or proteasome will likely increase healthspan and delay ageing [9].

For a long period, up to the 1960’s, the structural studies of natural products (NPs) served as the principle driving force for drug discovery and still constitute a unique source for novel leads and pharmacophores for medicinal chemistry. More than 50% of all new chemical entities (NCEs) introduced into pharmacotherapy over the period 1981–2010 derive (directly or indirectly) from NPs [10]. One critical reason for this output is their significant structural advantages due to their complexity and uniqueness [11]. Up to now, approximately 200,000 NPs were identified in plants and it is expected that the actual number will exceed 500,000 [12]. Nutrition and, specifically, regional dietary regimes, offer access to this NPs chemodiversity. Dietary plants, as medicinal plants, contain NPs that demonstrate powerful antioxidant properties and various other biological activities [13]. Numerous studies show that nutritional plant-derived compounds are involved in several physiological processes and have been found to possess significant beneficial effects on human health. These properties have been attributed to the presence of phytonutrients and biologically active NPs that exhibit pleiotropic and, likely, therapeutic effects in humans [14].

One of the geographical areas where nutritional habits have drawn attention as a prototype of nutritional protection against age-related diseases is the Mediterranean region. The traditional Mediterranean diet and its sub-branch, the Cretan diet, is widely considered one of the most important factors for the healthiness and long-life expectancy of populations from Mediterranean regions. It first came to scientific attention with the seven countries study [15,16]. This study started at the end of the 1950s and included 12,763 middle-aged men (40–59 years of age) from 16 cohorts of eight nations of seven countries. The results showed that higher mortality rates from coronary heart disease and other cardiovascular diseases (CVDs) were observed in North America and northern Europe, while lower rates were found in Mediterranean countries of southern Europe. These findings were strongly associated with the so-called Mediterranean diet. Recent studies have confirmed these findings [17,18,19,20,21,22,23,24].

The main characteristics of the Mediterranean diet are high consumption of unprocessed plant foods (vegetables, legumes, fruits, grains, nuts/seeds, and olive oil), moderate consumption of fish and red wine, and low consumption of dairy products, eggs, and meat. Wild and semi-cultivated leafy vegetables hold a key position in the daily diet. Many of these plants are cultivated and consumed widely, thus increasing the daily intake of antioxidants and other NPs by the population that adheres to the traditional diet with a potential benefit of protection against ageing and age-related diseases. However, while the “classical” concept of the Mediterranean diet has been a subject of numerous studies [25], the health impact of wild or semi-wild plants embedded in the local eating culture of a smaller region has been largely overlooked. Often, the consumption of specific plants is associated with information on a potential therapeutic effect based on folk medicine, a fact that is often disregarded when it comes to nutritional plants. Those local plants often contain high amounts of bioactive NPs such as polyphenols, carotenoids, terpenoids, polyunsaturated fatty acids etc. that could mediate the beneficial effects associated with their consumption. More than two hundred wild plants are consumed in the Mediterranean daily diet, yet, the majority of them have undergone limited studies regarding their chemical structure or the biological potential and activity of their content in phytonutrients [26,27]. For example, plant species belonging to the Asteraceae family such as *Cichorium spinosum*, *C. intybus*, and *Sonchus oleraceus* are consumed raw or slightly cooked on a daily basis in some cases, providing a range of polyphenols with strong antioxidant potential [28]. Edible legumes of the family Fabaceae (*Lotus tetragonolobus*, *Lathyrus ochrus*, *Vicia faba*, *Ceratonia* sp.) [29] are recommended twice a week for their nutritional value and their anti-hypercholesterolemic and anti-hypertensive effects [30]. Exotic fruits like *Opuntia ficus-indica* (Cactaceae) are reported to exhibit positive effects against type-2 diabetes [31] and diet-induced obesity [32]. *Olea europaea* (Oleaceae) and olive oil, which is an essential component of the Mediterranean diet, have multiple beneficial effects on enzymes involved in diabetes mellitus [33].

In addition to the Mediterranean diet, a well-known European dietary regime, global ethnic cuisines and dietary habits present similar interest in the consumption of plants with health benefits. Tunisia, belonging to the Mediterranean basin, shares similarities in the dietary habits, such as the high consumption of fruits and vegetables. For example, the date (*Phoenix dactilifera*, Arecaceae), a well-known Tunisian food plant, has been proven to have a hepatoprotective effect in vivo [34] and is traditionally used in Egypt for managing diabetes. The pomegranate (*Punica granatum*, Lythraceae), consumed raw and juiced, contains flavonoids that contribute to the amelioration of early diabetic nephropathy and glucose homeostasis [35]. In South Africa, tropical wild fruits are consumed on a daily basis. *Sclerocarya birrea* (Anacadiaceae), a fruit similar to mango, has great antioxidant and in vitro antidiabetic properties [36], *Carpobrotus edulis* (Aizoaceae) has been used as a traditional remedy against diabetes mellitus [37], and *Adansonia digitata* (Bombacaceae), the known baobab tree, provides fruits extremely rich in antioxidants and reduces glycemic response [38]. Furthermore, Eastern cuisines like Vietnam’s, use the edible leaves of *Perilla frutescens* (Lamiaceae) in stews, a plant that has shown an inhibitory activity against tumor necrosis factor alpha (TNF-α) [39]. Another example is the use of *Gnetum* sp. (Gnetaceae), a promising vascular protective agent, for the preparation of traditional curries [40]. *Houttuynia cordata* (Saururaceae), a plant with antihyperglycemic properties, is widely used as a raw garnish [41]. Moreover, in Chile, the consumption of native fruits is widespread. Two indigenous Chilean plants, the “Chilean guava” or *Ugni molinae* (Myrtaceae) and the “Chilean wineberry” or *Aristotelia chilensis* (Eleocarpaceae), are used for the preparation of jams or as food supplements due to their antioxidant properties [42,43,44,45,46,47], while *Lampaya* sp. (Verbenaceae), an indigenous Andes shrub, is used for liver and kidney damage [48].

However, it is impressive that even if the beneficial effects of healthy eating are greatly recognized and globally acknowledged, the majority of food plants that comprise the ingredients of the most established dietary patterns have not been investigated in depth and consequently the active constituents remain largely unknown. Along these lines, many currently used nutraceuticals or dietary supplements are based on NPs and are used in a broad, and from today’s perspective, rather unspecific way. More importantly, numerous health claims on plant-based products have been issued based on traditional use and eating habits. Nevertheless, those claims are in many cases not clearly associated with distinct bioactive NPs or well chemically characterized extracts and biological activity in vivo.

Despite the proven track record of NPs for their health-promoting properties and their noteworthy structural diversity, major drawbacks hinder their exploitation and further development. One of the most important bottlenecks is the complicated and time-consuming process required for tracing the active molecules. Valuable experimental time is wasted in laborious isolation processes with a high risk of “re-discovery” of an already known or inactive NP. For these purposes, some new advanced approaches have been proposed to tackle these obstacles. In particular, a new approach combining the traditional bio-guided fractionation along with modern analytical platforms has been developed. It correlates high-performance liquid chromatography (HPLC)-based (micro)-fractionation of plant extracts and activity determination on-line or at-line and enables an efficient localization of bioactive NPs directly in crude extracts. The obtained data can direct large-scale isolation steps to only metabolites of high interest [49]. In parallel, early metabolite identification of known NPs (dereplication) is performed with state-of-the-art analytical techniques [liquid chromatography (LC) coupled with mass spectrometry (MS) or high resolution mass spectrometry (HRMS), micro- and cryoprobe-nuclear magnetic resonance (NMR) methods, and LC coupled with solid phase extraction (SPE) and NMR (LC-SPE-NMR)] to assist scientists for directing the isolation only on NPs of interest and avoid rediscovery of compounds with previously reported activities [50]. In addition, rational medicinal chemistry approaches based on in silico chemical space calculation can be used to discover NPs with closely related structural features. Quantitative structure–activity relationship (QSAR) studies can be performed for finding optimum activity vs. toxicity profiles. Under the same concept, in silico screening techniques can offer a computational alternative that aims at the simplification and rationalization of the bioassays in order to increase the hit rate over the randomized approach [51]. This latter new strategy continuously gains in reliability and is nowadays recognized as a highly robust and efficient technique for the discovery of new leads [52]. By incorporating such approaches, the discovery procedure will efficiently be focused on NPs displaying the most favored chemical space, thus minimizing the isolation of unwanted-inactive compounds.

In the search for new biologically active substances, targeted bioassays still play an important role. Nevertheless, despite the technological breakthrough that NPs drug discovery experiences and in spite of enormous efforts and investments, many molecules active in vitro fail when tested in vivo. This is mainly due to limited bioavailability and metabolization processes (mostly under conditions of administration via the enteral route) before the molecules reach their target. Intestinal absorption, hepatic metabolism, distribution in body tissues, bioconversion by the colonic microbiota with formation of colonic metabolites, and elimination are main efficacy influencing factors. This is a very important drawback which, despite the impressive progress of the last years, does not allow the rationalization and the effectiveness of NPs discovery process. In addition, it is frequently observed that traditional medicinal plant extracts with a high level of ethnopharmacological and even clinical evidence, fail to show cell-based activity. The seeming “inactivity” of many NPs isolated from plants is likely caused because those are often pro-drugs, e.g., glycosides, which must undergo in vivo metabolic conversion (activation) [53,54]. Some well-known examples include the metabolic activation by gut bacteria of the isoflavone daidzein to the active metabolite equol, or of the phenolic compound salicin from willow bark to salicylic acid [55]. Metabolization assays mimic the metabolization of compounds in the human body, in particular, the gastro-intestinal dialysis model (GIDM) including microbial fermentation in the colon [56,57,58]. It is important to highlight that although these methods are used in pharmacological research, they have so far not been used in NPs research.

Therefore, the overall aim of the MediHealth project is to introduce a novel approach for the discovery of active agents in food plants from the Mediterranean diet and other global sources to promote healthy ageing. The MediHealth project incorporates new technologies and methods in NPs discovery and isolation in order to discover natural entities from Mediterranean and global dietary plants, promoting healthy ageing. The focus lies on two key aspects, namely accumulating ROS elicited damages, as well as the development of age-related metabolic diseases. A new approach is introduced by the ab initio parallel in vivo and in vitro testing and metabolization study of extracts and constituents thereof, in combination with sophisticated analytical techniques in a successive and integrated manner. MediHealth aspires to bring, based on scientific evidences, novel nutraceutical products and/or phytotherapeutics to the stage of development. This proceeds through a highly collaborative network between industrial and academic partners from European and non-European countries providing complementary scientific expertise, transfer of knowledge, and training of personnel.

## 2. Scientific Approach of the MediHealth Project

The MediHealth project comprises a unique combination of strategies including the following aspects:

- Investigation of food plants from the Mediterranean diet as well as from other global origins (e.g., Eastern Asia, North and South Africa, South America) based on established health benefits from traditional use, literature, and chemodiversity information.
- Introduction of a novel approach to assess pharmacological properties of metabolites derived from plant extracts based on newly developed extract metabolization protocols that mimic the processing of plant extracts by the human gut and liver as a prerequisite for meaningful in vitro testing.
- Use of a sophisticated and integrated concept combining extract metabolization protocols with advanced cell-based assays (focusing in particular on Nox4, Nrf2/Keap1, and proteasome activity modulation, as well as anti-diabetic effects) and their complementation by innovative tests for ageing and age-associated diseases in flies and mice, respectively, to identify natural entities promoting healthy ageing.
- Incorporation of state-of–the-art analytical techniques (e.g., microprobe-NMR, LC-SPE-NMR, LC-HRMSn), dereplication, and metabolomics strategies for the characterization of active compounds.
- Generation of solid data based on experimental scientific findings for the development of a new competitive product in the area of nutraceuticals, dietary supplements, and/or phytotherapeutics.

**Figure 1:**
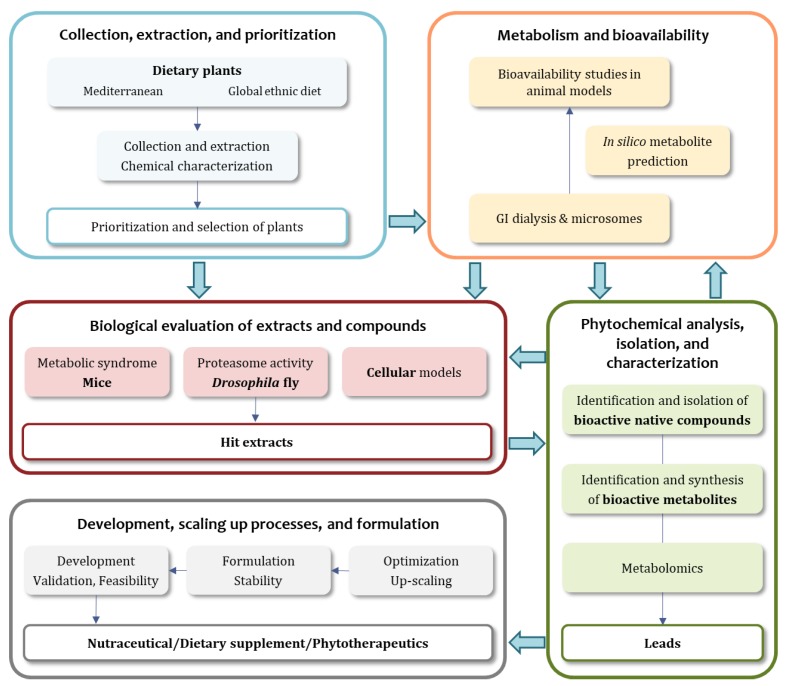
Workflow of the MediHealth project.

The main goal of the MediHealth project is to introduce a new concept for the discovery of small molecules in foods plants from the Mediterranean diet and other global sources with activity on healthy ageing and mainly metabolic disorders. This is achieved by an integrated and multidisciplinary approach and based on a well-organized workflow (Figure 1).

The first step of this approach is the collection of food plants with potential anti-ageing activity from diverse geographic regions with special attention to the Mediterranean diet. Interestingly, there is a huge number of food plants that are known to contribute to healthy ageing and to have beneficial effects on metabolic disorders but the available data on their active constituents are scarce. The selection of the plants is carried out mainly based on ethnopharmacological knowledge and literature information. Plant collection is followed by extraction and characterization using metabolic profiling. To lower the complexity of the experimental settings, in the course of this project, neither seasonal variations in the secondary metabolite profiles of plants or the influence of other environmental factors nor possible additive or synergistic effects of the simultaneous intake of two or more plants, which could happen in a complete diet, are considered. The chemodiversity data, including the analysis of the main chemical groups of substances, the complexity, and quantitative and qualitative aspects, facilitates the prioritization and the rational selection of promising plant extracts to be further investigated.

These candidate extracts are subjected to comprehensive functional assessment in an integrated in vivo/cell-based/metabolism experimental platform that comprises one of the most innovative aspects of the project. Specifically, in the context of MediHealth, a three-pronged approach to identify novel agents able to reduce ageing associated diseases and to improve healthspan is applied. To this end, the selected plant extracts are tested for potential health benefits in in vivo experiments in mice and flies. In parallel, they are also profiled in advanced cellular assays. In addition to cellular, fly, and mouse models, the same extracts are subjected to metabolization studies since plant extracts do not reach human tissues in their native form in most cases. These studies are crucial for the identification of active principles while offering more complete and meaningful information regarding their mechanism of action and the pharmacological potential of the plants. A human-like in vitro metabolization system, i.e., the simulation of gastrointestinal and microsomal metabolization is incorporated. In order to identify metabolites of a given plant extract that are active in the human body, plant extracts are metabolized in a GIDM including a gastric, an intestinal, and a colonic phase (where microbial conversions take place), simulating human gastro-intestinal metabolization. In addition, the dialysates containing the gastro-intestinal metabolization products are treated with hepatic S9 fractions to mimic liver metabolization. Plant extracts metabolized in this way are suitable for testing in cell-based assays. Metabolites generated by the in vitro model are also compared with metabolites actually detected in vivo in mice. In addition, the results obtained in this way are independently verified by the prediction of metabolites from genuine plant constituents using in silico tools. To our knowledge, these methods have so far not been used in NPs research.

For the cell-based testing, advanced bioassays for medium-scale screening procedures are used to measure biological effects related to ageing processes. Thus, isolated natural agents from dietary plants are evaluated for their bioactivities against Nox4 and intracellular ROS, the Nrf2/Keap1 signaling pathway, and proteasome activity in living cells. In the context of in vivo evaluation, a second approach is focused specifically on the development and alleviation of age-associated metabolic disorders, and is implemented by direct intervention studies in mice. To this end, mice are fed with extracts and multiple markers for the development of age-associated metabolic disorders are quantified, focusing specifically on dyslipidemia, type 2 diabetes, and CVD markers. In parallel, the capacity of the extracts to protect the proteome of the *Drosophila* fly from oxidative modifications (a major output of ageing) is examined by measuring the suppression of proteome oxidative damage as well as the activation of proteasome catalytic activity and the Nrf2 pathway. This triple approach including extensive in vivo and metabolic evaluation, combined with advanced cell-based testing in the early stages of plant extract evaluation is unique and offers valuable results that are expected to facilitate and substantiate the development of an agent that is safe, well characterized, and exhibits specific biological activity against age-related oxidative stress and metabolic disorders. Based on the results of these investigations, the derived data are correlated and analyzed, then the most promising plant extracts (hit extracts) are prioritized and forwarded to the next analyses.

The hit extracts (prior and after metabolization) are subjected to phytochemical analysis, isolation, and characterization of their chemical constituents. Bioactivity-guided (micro-) fractionation of native and metabolized extracts is incorporated for the isolation and structure elucidation of active compounds. State-of-the-art analytical techniques such as HPLC/ultra-high performance liquid chromatography (UHPLC) coupled with photodiode array detector (PDA), LC-HRMS, and LC-NMR are used for the identification of metabolites. The impact of hyphenated techniques has opened entirely new possibilities for the characterization of NPs in complex matrices such as extracts while instrumentation developments have also occurred in spectroscopic identification (LC-SPE-NMR, cryoprobe-NMR, and micro-NMR). This is complemented by the synthesis of selected bioactive metabolites that might be necessary for verification of the chemical structures, as well as qualitative and quantitative aspects of the observed bioactivity. Finally, the identified active molecules (both precursors and metabolites) are subjected to in silico virtual screening against a panel of relevant pharmacological targets offering better insight into the possible mechanism of action and suggestions for likely additional activities.

According to the derived results, metabolomic approaches are applied to different batches of the hit extracts or parent plant materials in order to identify the optimal sources for the procurement of the plants of interest (period of collection, close related species or genera containing similar profile of bioactive compounds). This defines the lead extracts to proceed with up-scaling and development procedures. To achieve the optimal procedure for metabolite procurement, optimization of extraction focusing on “green” extraction techniques and scaling-up procedures are applied. Based on this, a pilot scale production of the lead extracts and/or enriched extracts are developed while pre-formulation, final product characterization, and stability studies are also performed. To carry the lead agents (extracts, enriched extracts, or compounds) to the stage of development, feasibility studies also take place. It is important to underline that the whole research initiative needs to be compliant with local and international guidelines and frameworks related to bioprospecting and the protection of indigenous resources.

MediHealth is a project with high scientific goals that aspires to introduce a new approach and pipeline in modern NPs drug discovery process. Along these lines of research, it is obvious that a multidisciplinary consortium is needed to successfully implement such a demanding and ambitious project. Therefore, MediHealth has brought together fifteen different groups of experts from both academia and industry, taking into consideration their competences, complementation, and knowledge but primarily the scientific needs of the project. The MediHealth network is based on three basic levels of expertise, i.e., chemistry (phytochemistry, analytical chemistry, organic chemistry, and medicinal chemistry), biology (pharmacology, molecular biology, cell biology, metabolism, and biochemistry), and product development (production, quality control, feasibility and stability studies, and marketing). Specifically, MediHealth has gathered an impressive consortium of experts on an international level that is comprised of seven academic groups from European countries (Austria, Belgium, Greece, and Switzerland), four European industrial participants (from Austria, France, Germany, and Greece), and four academic partners from non-European countries (Chile, South Africa, Tunisia, and Vietnam). In addition to the pure experimental and scientific goals of the project, valuable knowledge and know-how is exchanged for the benefit of the researchers and experts involved but also between academia and industry, as well as between European and non-European countries. Furthermore, due to the high degree of expertise and complementarity, meaningful interactions are evolved in the area of NP chemistry, analytical chemistry, biology and pharmacology, metabolization studies, metabolomics, separation and large-scale isolation, synthesis, and product development. Using such an interdisciplinary novel approach increases significantly the chances of successfully identifying NPs that can be used to treat age-associated diseases.

## 3. Organization of the MediHealth Project

The scientific tasks of the MediHealth project can be assigned to five major project parts, as depicted in Figure 1, i.e., the collection, extraction, and prioritization of dietary plants, the investigation of metabolism and bioavailability aspects, the biological evaluation in mice, flies, and cellular models, the phytochemical analysis, isolation, and characterization, and finally product development, scaling-up processes, and formulation.

### 3.1. Collection, Extraction, and Prioritization of Dietary Plant Species with Possible Healthy Ageing Effects

In the beginning of the project, dietary plants from the Mediterranean diet and other sources with potential anti-ageing profiles are rationally selected. Therefore, based on data from literature and knowledge about traditional use, a series of plant species from the Mediterranean (e.g., Cretan) diet and edible plants used in other areas with potential healthy ageing effects is collected. A small quantity of the plant species (ca. 5 g dried and ground material, respectively) is extracted using an appropriate method (e.g., accelerated solvent extraction, ASE) and solvent (e.g., ethanol, water), respectively. The derived extracts are phytochemically characterized using thin layer chromatography (TLC) and HPLC fingerprint analysis in combination with MS. Finally, they are prioritized based on chemodiversity, ethnopharmacology, traditional use, as well as additional criteria such as plant availability and capability of cultivation or sustainable collection. A selection of plant extracts with diverse chemical profiles and healthy ageing potential is forwarded to the in vitro GIDM as well as to biological evaluation in the in vivo mouse and fly models.

### 3.2. Investigation of Metabolism and Bioavailability Aspects of Selected Plant Extracts and Constituents

Given the fact that most of the compounds present in plant extracts will most likely be metabolized, extracts of the selected plants are subjected to in vitro simulation of gastro-intestinal metabolization, followed by treatment with microsomal S9 fractions to mimic liver metabolization in humans. For this purpose, an in vitro GIDM, including microbial fermentation in the colon, is used, to mimic human metabolization processes and produce metabolites of native plant constituents. The GIDM simulates gastric and intestinal physiological conditions, including digestion pH, digestion time, a pepsin solution for the stomach, gradual pH change from stomach to intestine, a pancreatin-bile extract in the small intestine, body temperature, and an anaerobic environment. The intestinal stage is performed using stirred cells, equipped with a dialysis membrane (MW cut-off 1000 Da) and a dialysis bag. The microbial compartment of the colonic phase is simulated using cultures of fecal slurry (homogenized human stool sample). The continuous removal of the dialyzed components through a dialysis membrane simulates the one-way pathway in the gastro-intestinal tract from lumen to mucosa. This model helps considerably in resolving the mechanism of metabolization and the identification of active principles in complex mixtures such as plant extracts and food materials. For each native plant extract, 6-10 dialysates are collected at different times in the gastric, intestinal, and colonic compartments. While part of each dialysate is forwarded to in vitro pharmacological evaluation, part of it is also studied using a liver metabolization assay, since also in the human body, further metabolization reactions would occur after absorption, mainly in the liver. In specific, the samples are analyzed using microsomal S9 fractions (commercially available; prepared from liver), which consist of cytosol and microsomes. After incubation with S9 fractions, the reaction mixture is treated with sulfatase/glucuronidase enzymes to hydrolyze the formed conjugates. The derived samples are subjected to in vitro pharmacological investigation.

After comparing the results of the in vitro analysis of the extracts metabolized in the gastro-intestinal filter, prior to and after liver metabolization, with activities of the genuine extracts observed in the in vivo models, hit plants are selected. Metabolites of these hit plants generated by the in vitro GIDM and the treatment with S9 fractions, are identified by chromatographic and spectroscopic tools. In silico prediction methods are also used to facilitate the identification of derived metabolites. In particular, bioactivity profiler software (e.g., PASS online) is employed to predict likely interactions between established genuine plant constituents and metabolic enzymes. Furthermore, software tools (e.g., FAME or Metabolizer) are used to predict the structure of metabolites formed from the original plant constituents.

Moreover, for selected lead extracts that show positive results in the in vivo mouse model, bioavailability studies in mouse models are performed. Blood samples are evaluated by NMR and LC-HRMS using two complementary approaches: (i) targeted analysis for checking the presence of plant metabolites and (ii) untargeted analysis for measuring the changes in endogenous metabolites related to phenotypic effects. The serum metabolites present in mice are also compared with the metabolites generated by the GIDM and the treatment with S9 fractions.

### 3.3. Biological Evaluation of Extracts, Fractions, and Compounds

Another important part of the MediHealth strategy is the biological evaluation of extracts, fractions, isolated constituents and metabolites thereof, as well as (semi-) synthetic analogues. To identify food plants that have the capacity to induce beneficial metabolic changes, a two-pronged approach is performed, where animal models for ageing and the development of age-related diseases are complemented by cell-based assays.

#### 3.3.1. Analysis in Mouse Models for the Development of Age-Related Diseases

In order to investigate the potential of selected food plant extracts to delay ageing, C57BL/6 mice fed either a control diet or a diabetogenic high fat diet are treated for two weeks with crude plant extracts or vehicle. Metabolic markers related to age-dependent diseases are measured, such as plasma glucose, total triglycerides, free fatty acids, and cholesterol, as well as a detailed plasma lipoprotein profile. In addition, insulin sensitivity is determined as a surrogate marker for the development of type 2 diabetes. Since age-related diseases have been linked to a decrease in basal energy consumption, energy combustion by heat production in response to food plant extract treatment is also determined by analyzing body core temperature profiles and metabolic activity during the course of dietary interventions. On a cellular level, liver, muscle, and adipose tissue as key metabolic organs are studied for changes in the expression of key factors involved in the progression of age-related diseases. These experiments are tightly linked to the analyses in *Drosophila* flies and in cellular models to harmonize the in vitro and in vivo studies for the analysis of ROS mediated damage to tissues that could influence the progression of age-related diseases.

Out of the biologically investigated extracts, hit extracts are selected for further analysis, including fractionation and isolation of compounds. Selected fractions generated in this way and their isolated components are tested in the model described above and also for the ability to improve metabolic parameters in leptin-deficient *ob*/*ob* mice as well as hyperlipidemic apolipoprotein A5-deficient mice. Taken together, these experiments show whether selected food plants have the power to positively influence body weight and the metabolic profile in extreme models of obesity and hyperlipidemia.

#### 3.3.2. Analysis in a *Drosophila* Fly Model for the Development of Age-Related Diseases

In order to investigate the potential of selected extracts derived from dietary plants to delay ageing in flies, their capacity to protect the proteome of *Drosophila* flies from oxidative modifications and to activate in vivo the Nrf2/Keap1 pathway and/or the proteasome peptidase activities are evaluated.

The hit extracts (selected on the basis of results of the pharmacological investigation of extracts in mice, flies, and cellular models) are analyzed further, including fractionation and isolation. Selected fractions and their isolated components are tested in the fly in vivo experimental platform for:

- Suppression of proteome oxidative damage. *Drosophila* flies are fed with standard food containing the selected NPs under study and the proteome oxidative damage is assayed by measuring proteins’ carbonyl groups.
- Activation of the Nrf2 signaling pathway. Nrf2 activation is assayed by analyzing the gene expression levels of its transcriptional targets, namely antioxidant and proteasome genes.
- Activation of the proteasome catalytic activity. The major hydrolyzing enzymatic activities of the proteasome are measured by adding specific fluorogenic peptides (substrates) in lysates of flies’ somatic tissues.
  For the most effective samples, longevity assays in large numbers of *Drosophila* flies are performed in order to reveal potential in vivo healthspan increasing and/or anti-ageing effects. Extensive statistical analyses in these large cohorts couple these analyses.

#### 3.3.3. Analysis in Cellular Models for Antioxidant Signaling

Innovative cellular models related to the evaluation of agents with anti-ageing profile are used. The selected extracts, before and after metabolization, are assayed for their effects on Nox4 activity (e.g., RPTEC/TERT1 or HEK293 cells expressing a Nox4 transgene), proteasome activity (e.g., HDF/TERT1 expressing GFP-degron), for their ability to activate the Nrf2/Keap1 antioxidant signaling pathway (e.g., transgenic human RPTEC/TERT1 and HEPTEC/TERT1 carrying the human Nrf2 or Keap1 gene promoter cloned upstream of the firefly luciferase gene), and for anti-diabetic activity such as adipocyte differentiation in 3T3-L1 cells, measuring triglycerides accumulation and differentiation markers. In addition, the same samples are used to determine the concentration range in which no cytotoxicity is observed. This is measured by using the 3-(4,5-dimethylthiazol-2-yl)-2,5-diphenyltetrazolium bromide (ΜΤΤ) method. These experiments contribute to the prioritization of extracts based on the in vivo assays in mice and flies. Fractions derived from extracts of the hit plants, their isolated constituents, as well as synthesized metabolites are also tested in the cell-based model systems described above. The most active samples are forwarded to more extended biological investigations, including the evaluation of their ability to delay cellular senescence in human umbilical vein endothelial cells (HUVECs) and to prevent UVB-induced senescence of human diploid fibroblasts.

The hit plants are also subjected to activity profiling with in silico virtual screening. In particular, the 3D structures of identified active native and metabolized constituents of the hit plants are stored alongside the bioactivity data generated in the cell-based bioassays. These data in combination with a set of in silico target prediction methods, such as pharmacophore modelling and the similarity ensemble approach (SEA), are used to identify potential molecular targets and mechanisms of action for the active (native and metabolized) plant constituents. Experimentally validated pharmacophore models and target-based assay methods for a range of individual targets are used. Through this process, active scaffolds for the selected biological targets are determined and principle structure-activity relationships (SARs) are defined.

### 3.4. Phytochemical Analysis, Isolation, and Characterization

The bioactive native and metabolized hit extracts are fractionated in a bioactivity guided way, supported by data from the in vitro (mainly metabolized extracts) and in vivo systems (only native extracts), leading to the isolation and purification of the bioactive compounds. Isolation and purification of bioactive plant constituents from native extracts is performed on a preparative scale using state-of-the-art preparative chromatographic separation techniques, e.g., flash or medium pressure liquid chromatography (MPLC) or fast centrifugal partition chromatography (FCPC). The bioactive plant constituents are identified using state-of-the-art spectroscopic techniques including UV-Vis, infrared radiation (IR), circular dichroism (CD), 1D- and 2D-NMR, high resolution NMR equipped with microflow, microprobes, and cryoprobes, as well as HRMS based on time-of-flight or orbital traps technology. The same methods are used to assess the purity of the isolated NPs. An aliquot of the isolated bioactive plant constituents is sent to in vitro metabolization.

Active metabolized extracts of the hit plants are submitted to bioactivity guided LC-micro-fractionation in 96-well plates. Bioactive micro-fractions are analyzed by UHPLC coupled with a photodiode array detector (PDA), evaporative light scattering detector (ELSD), and HRMS, in combination with NMR techniques for the identification of the bioactive metabolites. In silico prediction tools are also used to support the identification procedure. Selected bioactive metabolites and structural analogues are (semi-) synthesized using state-of-the-art strategies.

Pure bioactive plant constituents and metabolites are subjected to comprehensive pharmacological analysis in vitro (mainly metabolites) and in vivo (only native plant constituents). Based on the results of the detailed phytochemical and pharmacological investigations, lead compounds, extracts, or enriched extracts are identified. Innovative metabolomics strategies are applied for the identification of the optimal plant sources of the leads with respect to industrial needs.

### 3.5. Development, Scaling Up Processes, and Formulation

To carry the lead agents (extracts, enriched extracts, or compounds) to the stage of development of nutraceuticals or phytotherapeutic agents, optimization and application of up-scaling procedures are carried out. For the optimization of the extraction and isolation methods, several parameters are taken into consideration (e.g., effectiveness, robustness, reproducibility, and scalability to the industrial level). The “green” extraction techniques of supercritical-fluid-extraction-CO_2_ (SFE-CO_2_) and subcritical water extraction (SWE) are employed and compared to more conventional techniques. For the development and the optimization of the SFE-CO_2_ extraction methods, special attention is given to CO_2_ pressure, extractor and collector temperature, CO_2_ flow, CO_2_/starting material ratio, and the percentage of co-solvent (ethanol, water, or mixtures thereof). Furthermore, in order to determine the optimal conditions for SWE extraction, the effects of varying parameters (e.g., extraction temperature and duration) are investigated. Using analytical techniques such as near infrared spectroscopy (NIR) and MS, the obtained extracts can be directly compared and optimal conditions can be identified. For implementation of a fast and cost-effective quality control method for the produced extracts, the data acquired in the analytical screening is used to create an NIR-based model for the determination of extract quality. If a final purification step is needed, this is performed using the centrifugal partition chromatography (CPC) technology. In this case, industrialization processes in CPC seeking optimal conditions for scale-up are considered. Moreover, adsorption resins technology (ART) is used mainly for the production of enriched extracts while spray drying techniques are also incorporated. A series of different resin types (e.g., XAD4, XAD16, and XAD-7HP) are evaluated for the optimal capacity and selectivity. Furthermore, to provide a smooth transition into industrial implementation and commercialization, all the respective steps of the process as well as the corresponding results are evaluated for their compliance with the demands of industry and regulatory authorities.

In addition, according to their chemical characteristics, the lead agents are assayed using different formulation concepts, with the intention of the development of nutraceuticals and phytotherapeutic agents. For instance, lyophilization and the use of appropriate formulatory agents are encapsulated following the main formulation processes, i.e., extrusion spheronization, solution/suspension layering, powder layering, wet granulation pelletizing, and melt granulation pelletizing.

For the formulated lead products, analytical methods for marker components are developed and validated according to the ICH guidelines, including linearity, repeatability (intermediate precision and repeatability on different concentration levels), and accuracy. Based on the respective characteristics and analytical demands of the products, different analytical platforms are incorporated (e.g., UPLC-DAD, HPLC-ELSD, and LC-MS). The microbiological quality of the lead products is also assessed.

Moreover, following the development process, stability studies are performed on the lead products under storage conditions in accordance with ICH guidelines using chromatographic techniques. Multiple parameters, such as temperature, humidity, and pH range, are investigated (e.g., 25 °C, 60% RH; 30 °C, 65% RH; 40 °C, 75% RH).

Finally, to determine the commercialization potential of the lead agents, feasibility studies are conducted. These include a detailed market analysis and the investigation of economic prospects and commercial exploitation of the potential product.

## 4. Conclusions

The MediHealth project introduces an innovative concept for the investigation and exploitation of dietary plants incorporating an integrated approach combining in silico, cell-based, in vivo, metabolization studies, and state-of-the-art techniques. It aspires to produce valuable and solid data supporting the development of novel nutraceuticals or phytotherapeutics to promote healthy ageing. We hope that sharing the strategy of this novel and unique multidisciplinary approach helps to increase the scientific interest in NPs research with special regard to the exploitation of diet regimes towards healthy ageing.

## Abbreviations

The following abbreviations are used in this manuscript:

AREs	antioxidant response elements
ART	adsorption resins technology
ASE	accelerated solvent extraction
CD	circular dichroism
CPC	centrifugal partition chromatography
CVD	cardiovascular disease
ELSD	evaporative light scattering detector
FCPC	fast centrifugal partition chromatography
GIDM	gastro-intestinal dialysis model
HPLC	high-performance liquid chromatography
HRMS	high resolution mass spectrometry
HUVEC	human umbilical vein endothelial cell
IR	infrared radiation
Keap1	Kelch-like ECH-associated protein 1
LC	liquid chromatography
MPLC	medium pressure liquid chromatography
MS	mass spectrometry
ΜΤΤ	3-(4,5-dimethylthiazol-2-yl)-2,5-diphenyltetrazolium bromide
NCEs	new chemical entities
NIR	near infrared spectroscopy
NMR	nuclear magnetic resonance
Nox4	NADPH oxidase 4
NPs	natural products
Nrf2	NF-E2-related factor-2
PDA	photodiode array detector
QSAR	quantitative structure–activity relationship
ROS	reactive oxygen species
SAR	structure-activity relationship
SEA	similarity ensemble approach
SFE-CO_2_	supercritical-fluid-extraction-CO_2_
SPE	solid phase extraction
SWE	subcritical water extraction
TLC	thin layer chromatography
UHPLC	ultra-high performance liquid chromatography
WHO	World Health Organization
